# Long-term Treatment Benefits and Prolonged Efficacy of OnabotulinumtoxinA in Patients Affected by Chronic Migraine and Medication Overuse Headache over 3 Years of Therapy

**DOI:** 10.3389/fneur.2017.00586

**Published:** 2017-11-03

**Authors:** Simona Guerzoni, Lanfranco Pellesi, Carlo Baraldi, Michela Maria Cainazzo, Andrea Negro, Paolo Martelletti, Luigi Alberto Pini

**Affiliations:** ^1^Headache and Drug Abuse Research Centre, Policlinico Hospital, Department of Diagnostic Medicine, Clinical and Public Health, University of Modena e Reggio Emilia, Modena, Italy; ^2^Regional Referral Headache Centre, Sant’Andrea Hospital, Department of Clinical and Molecular Medicine, Sapienza University, Rome, Italy; ^3^Center for Neuroscience and Neurotechnology, Department of Biomedical, Metabolic and Neural Sciences, University of Modena e Reggio Emilia, Modena, Italy

**Keywords:** chronic migraine, OnabotulinumtoxinA, long-term treatment, quality of life, tolerance, headache, medication overuse headache

## Abstract

**Background:**

Chronic migraine (CM) affects about the 2% of the general population and it has been recognized as one of the most-disabling conditions worldwide by the World Health Organization. CM is often associated with the overuse of abortive medication, which determines the worsening of headache itself and the development of a secondary headache called medication overuse headache. The management of these associated conditions is difficult, but a growing amount of evidence is pointing out the effectiveness and the good safety profile of OnabotulinumtoxinA (OnabotA). Despite this, data on OnabotA effects and safety in long-term use lack. The purpose of the present article is to retrospectively assess the efficacy and safety of OnabotA in a cohort of chronic migraineurs with drug overuse from the 18th month of treatment until the third year.

**Materials and methods:**

90 chronic migraineurs with medication overuse were enrolled between January 2013 and February 2017. All patients were treated with OnabotA according to PREEMPT dictates. Before every injection session the headache index, the analgesic consumption, the visual analog scale for pain score, the 36-items short form health survey questionnaire score, the 6-items headache impact test (HIT-6) score and the Zung self-rating anxiety and depression scale scores were collected. Adverse events were carefully registered. A simple linear regression was performed to explore the mean changes in the abovementioned parameters for a single injection session and mean comparison tests were performed using the one-way analysis of variance followed by Tukey–Kramer post-hoc test.

**Results:**

A significantly improvement for a single injection was registered for all the above-mentioned parameters. Headache index, analgesic consumption, visual analog pain scale, and 6-items HIT-6 scores were significantly lower than baseline from the 18th month of treatment onwards. The 36-items short form health survey questionnaire scores were significantly higher than baseline at every injections session from the 18th months onwards. Zung scales did not change. No serious adverse events were assessed and no adverse events-related drop-outs were seen.

**Conclusion:**

OnabotA effectiveness and safety last until 3 years of therapy, raising the possibility of the use of this therapy even for many years in CM prevention.

## Introduction

Chronic migraine (CM) is a common neurological disorder affecting about the 2% of the general population (1). It is characterized by over than 15 headache days per month, eight of which presenting migraine features, for at least 3 months (2). The recurrent and excruciating pain impacts negatively on patients’ quality of life: chronic migraineurs are more frequently depressed, less likely to be employed and have a lower socioeconomic status than episodic migraineurs (3). Besides this, they have also a higher rate of sanitary services use and, considering the prevalence of this disease, CM is one of the greatest causes of sanitary expenditure worldwide (4). Chronic migraineurs usually assume a large amount of symptomatic drugs to relief pain, with the 73% of them overusing abortive medications, mainly triptans and non-steroidal anti-inflammatory drugs (NSAIDs): this can paradoxically lead to the worsening of CM, generating a secondary headache called medication overuse headache (MOH) (2). MOH complicates CM management: besides headache worsening, patients are exposed to a greater likelihood of developing drugs-related AEs, mainly cardiovascular and gastrointestinal (5). Since this, the withdrawal of the overused drug is mandatory to reduce headache severity and increase preventive treatments effectiveness, which is even lower in patients affected by MOH than in chronic migraineurs without MOH (6). The higher therapeutic failure rate is not only due to the worse conditions of MOH-sufferers but also to the generally low adherence toward prescribed treatments (7): the reduction of treatment effectiveness along time and the incoming of drugs-related AEs usually force patients to discontinue preventive medications (8). Considering this, for a good therapeutic response is crucial to have a preventive therapy able to reduce pain with mild AEs, maintaining its effectiveness and safety on long-time periods (9). OnabotulinumtoxinA (OnabotA) effectiveness was clearly demonstrated from the results of the Phase III Research Evaluating Migraine Prophylaxis Therapy (PREEMPT) study (10–12). Results from the PREEMPT 1 and 2 trials outpointed a significant higher reduction than placebo in the number of headache and migraine days in the treated group at the 24th week of treatment. This was also reflected by a lower triptan consumption despite a similar analgesic intake (10, 11). Notably, OnabotA-treated group had a significant higher reduction than placebo group of the 6-items headache impact test (HIT-6) score at all time points. Moreover, a significantly lower number of patients with a HIT-6 score higher than 60, that is with a substantial headache impact on their quality of life, was seen in the treated group (12). Furthermore, all these studies outpointed the better safety profile of OnabotA, which gave a drop-out rate not significantly different than placebo (10–12). Even those articles comparing OnabotA with other preventive treatments outpoints that, besides a similar effectiveness, OnabotA better safety profile is associated with a lower discontinuation rate and should be chosen as first-line therapy to improve patients adherence (13). Despite this, it is still unclear if OnabotA effectiveness for CM disease lasts even after years and if its good safety profile is maintained through time (14). In a previous study, our group has already demonstrated the effectiveness and safety of OnabotA until the 18th month of treatment in chronic migraineurs complicated with MOH (15) and, to our knowledge, data regarding OnabotA effectiveness until 36 months are currently missing. The aim of this retrospective study is to explore the safety and the effectiveness of OnabotA, administered quarterly and following the PREEMPT paradigm (16), from the 18th to the 36th month of therapy in patients affected by CM complicated by MOH.

## Materials and Methods

### Patients Selection

In Modena University headache and drug abuse center, a retrospective study was performed on patients affected by CM complicated with MOH according to the International classification of headache disorders-third edition-beta version (ICHD-III-beta) (2) and received OnabotA injection as prophylaxis. At April 2016, 134 patients were in treatment with OnabotA. Forty-four patients were immediately excluded because their poor compliance in properly filling the headache diary. Ninety patients were considered eligible and enrolled between March 2016 and September 2017. Every patient failed, at least, three drug preventive treatments and underwent a hospital detoxification therapeutic recovery before start OnabotA injections, which were administered between January 2013 and February 2017. Injection procedures strictly followed PREEMPT protocol items: 155 U of OnabotA were regularly injected every 3 months in 31 sites of head, neck, and shoulders by an expert clinician (16). Patients were allowed to take abortive medications without any restriction, whereas no more than one oral preventive medication at a stable dose. If the dose and/or type of preventive treatment changed during OnabotA treatment, only data until the previous injection cycle were pooled in the analysis. However, patients could continue OnabotA injections under clinical judgement. If the patient did not find benefits from the canonical 155 U, the physician could adopt a “follow the pain strategy,” adding up to 40 additional units to the original dose into eight injection sites in temporalis, occipitalis, and/or trapezius muscles, as suggested by other authors (16). The study was approved by the Ethical Committee of Modena (protocol number 394/CE) and performed in observation of the latest version of the declaration of Helsinki.

### Procedures

During every injection session, the mean number of headache days over 30 days (MNHD), the mean number of abortive medications taken every day (analgesic consumption-AC) and the mean value of the visual analog scale for pain (VAS) score were collected from the headache diaries. HI, AC, and VAS score were considered as the primary end-points of the study.

Patients’ quality of life was assessed using the HIT-6 score, which has been specifically validated in patients with CM (17); in particular the Italian version of the HIT-6 was used (Version 1.1 ©2001 QualityMetric, Inc., and GlaxoSmithKline Group of Companies).

Mental and physical health were analyzed using the Italian version of the 36-items short form health survey questionnaire (SF-36) (18). Anxious and depressive symptoms were analyzed using the Zung self-rating anxiety scale (ZUNG-A) and the Zung self-rating depression scale (ZUNG-D), respectively (19). HIT-6, SF-36 mental and physical, ZUNG-A and ZUNG-D scores were considered as secondary end-points. The abovementioned questionnaires were filled by patients before every injection and explored the previous 3 months.

### Statistical Analysis

All continuous variables were expressed as mean ± SD, categorical ones were expressed as proportions and percentages. For all end-points, a simple linear regression model was built using the end-point itself as dependent variable and injection cycle number as the independent one. The slopes of abovementioned models were analyzed to explore the mean change of every outcome for a single injection session through time, in order to identify an average mean change of the explored parameters through time. After that, end-points means at every injections session from the 18th month onward were compared with baseline using the one-way analysis of variance followed by Tukey–Kramer *post hoc* comparison test, in order to define if the improvements previously achieved until the 18th months of treatment (15) were maintained even on further time-points. The influence of a coexistent preventive treatment on the explored outcomes was explored performing a two-way analysis of variance. Only first class preventive treatments in Italian guidelines for headaches (20) were considered, split for drug classes. Moreover, a Student’s *t*-test was performed for every end-point, comparing its mean at the seventh injection cycle (after 18 months of therapy) with the mean at the last one to better define the changes in the explored parameters between these checkpoints. All results were approximated at the second decimal figure; linear regression slopes exploring the mean changes of explored parameters for every injection session at the third. Statistical analysis was performed using the STATAIc 13.1 software.

## Results

### Demographic Analysis and Drop-Outs

The analyzed sample was composed by 90 patients, 14 men and 76 women, aged between 35 and 65 years (mean ± SD = 45.21 ± 10.12). The most overused drugs were triptans (71/90–78.89%) followed by NSAIDs (41/90–45.56%), while only three patients overused combination drugs (3–3.33%). Oral drugs were taken by the 88% of patients, the 20% used also intramuscular drugs and the 15% used also rectal formulations. Thirty-four patients used a first class preventive treatment other than OnabotA: 4 used anti-hypertensive drugs, 15 antidepressants, and 15 antiepileptics (20). No patients took simultaneously two first class preventive treatments. Patients who took anti-hypertensive drugs stopped them before the seventh injection cycle, due to inefficacy (three patients) and one adverse event (hypotension), so data from these patients were not pooled in the two-way analysis of variance. Eight patients underwent the 195 U treatment during no more than one injection cycle each. Eighty-eight out of 90 patients (97.8%) fulfilled the diagnostic criteria for CM at the beginning of the study. After the first year of treatment, patients suffering for CM were 37 out of 59 (62.72%), becoming the 66.67% at the second year (14 out of 21 patients) and the 53.85% at the third year (7 out 13 patients). The proportion of chronic migraineurs at the baseline is significantly lower than the ones at the first, second, and third year. Those ones were not significantly different (Fisher’s exact test, data not shown). All 88 chronic migraineurs at the beginning were also considerable as MOH-sufferers. After the first year of therapy their percentage decreased to the 59.32% (35 out of 59 patients). At the second year the proportion of MOH-sufferers increased at 13 out of 21 (61.9%) and at the third year became of 7 out of 13 patients (53.85%). MOH-sufferers proportion at the baseline was significantly higher than the ones at future time-points, but no significant differences were found between them (Fisher’s exact test, data not shown). 14 out of 90 patients (12.6%) reduced of at least the 50% the number of headache days after the first year of treatment; at the second year the percentage was the 11.11% (2/18) and at the third the 7.7% (1/13). Of the 90 patients enrolled, 24 changed the dose and/or type of preventive treatment other than OnabotA due to side effects and, even if they continued OnabotA injections, further data were not pooled in the analysis. One patient tried a muscular electric stimulator without consulting physicians and her data from that moment onward were not pooled in the analysis. Globally, 14 patients stopped OnabotA injection during the observation (14/90–15.56%): one patient decided to stop treatment because she was almost pain-free, three patients were lost at follow-up and 10 patients discontinued OnabotA due to lack of efficacy. No drop-outs were caused by OnabotA-related AEs. Globally, only patients who discontinued OnabotA treatment because of lack of benefit and those ones who were lost at follow-up were considered as drop-outs, giving an overall number of drop-outs of 13/90 (14.44%).

### Linear Regression Models for All End-Points

All linear regression models performed had a statistically significantly slope, indicating a significantly mean change over zero of the explored outcomes for single injection session. In particular, a significantly (*P* < 0.01) mean reduction in all primary end-points (MNHD, VAS, score and AC) at every injection session was noted. A general significantly (*P* < 0.01) reduction was also observed for the HIT-6, ZUNG-A and ZUNG-D scores. Moreover, a statistically significant (*P* < 0.01) increase was seen for the means of SF-36 scores, both mental and physical. All results are summarized in Table 1.

**Table 1:** Linear regression slopes and relative 95% CI.

End-point	Slope [95% CI]
Mean number of headache days over 30 days	−0.044 [−0.051 to −0.037]**
Visual analog scale for pain	−0.423 [−0.47 to −0.376]**
AC	−0.111 [−0.14 to −0.082]**
6-items headache impact test	−0.726 [−0.904 to −0.548]**
SF-36 P	2.106 [1.61 to 2.603]**
SF-36 M	2.205 [1.659 to 2.701]**
ZUNG-A	−0.714 [−0.954 to −0.473]**
ZUNG-D	−0.607 [−0.887 to −0.327]**

*P < 0.05, **P < 0.01.

*The significance refers to the Wald t-statistic test built up on models’ slopes to test the null hypothesis according with slope itself is not significantly different from 0*.

### One-Way Analysis of Variance for Primary and Secondary End-Points, Student’s *t*-Test between the Seventh and the Last Injection Cycle and Two-Way Analysis of Variance

**Figure 1:**
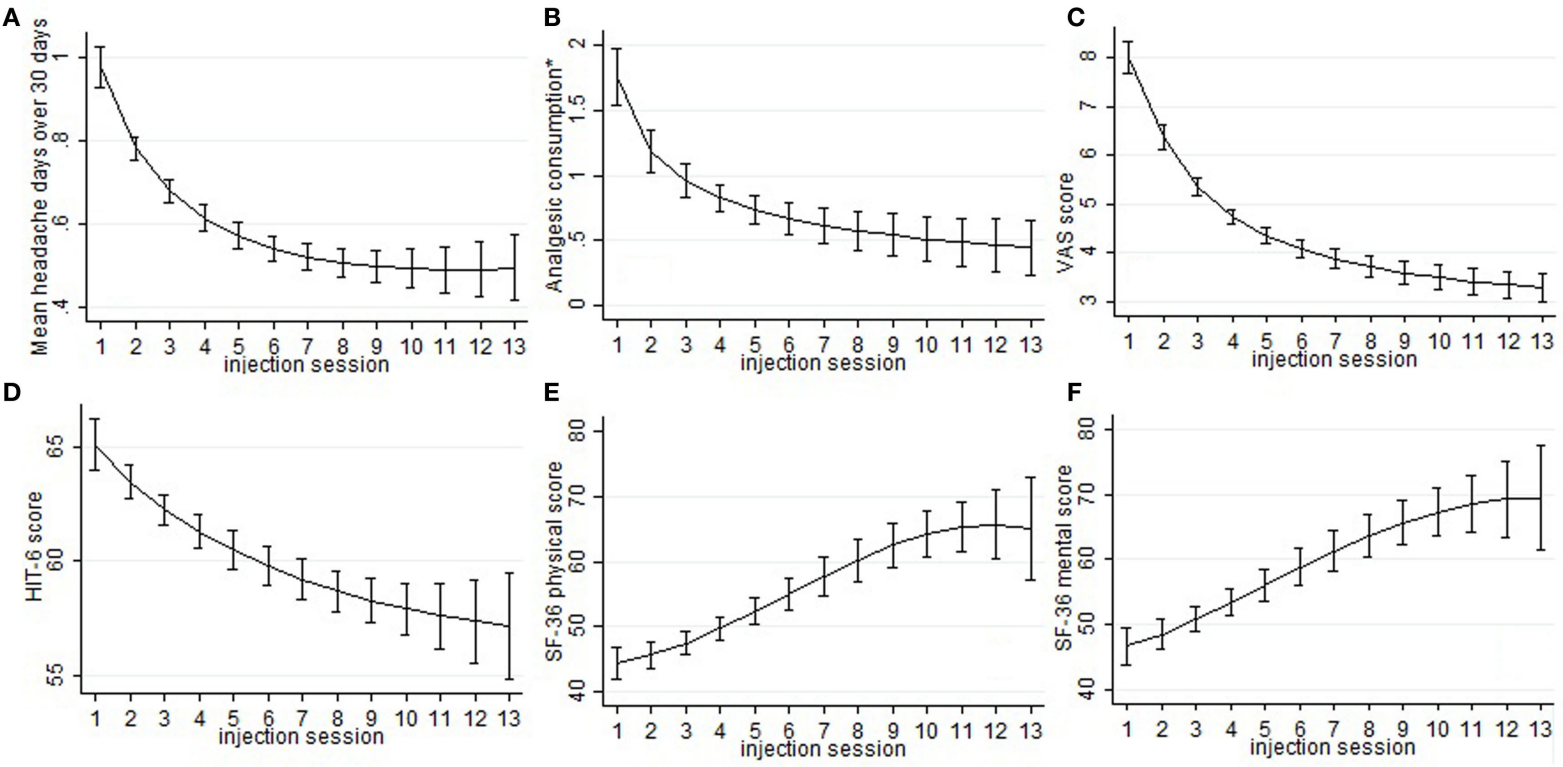
Sub-graphs indicate the means and the relative 95% confidence intervals of the explored parameters, despite the ZUNGA and ZUNGD scores, for every injection session. In particular, the trend of the following outcome has been represented: mean headache days over 30 days 492 (sub-graph A), mean number of abortive medications taken every day (sub-graph B), visual analog scale for pain score (sub-graph C), 6-items headache impact test score (sub-493 graph D), SF-36 physical score (sub-graph E), and SF-36 mental score (sub-graph F). *mean number of abortive medications taken every day.

Mean number of headache days over 30 days, CA, and VAS score means were significantly lower than baseline at every injection session from the 18th month of treatment onward, but not always lower than the previous time-point. The changes observed for MNHD and VAS scores were followed by similar ones for the AC, significantly lower than baseline at all time-points from the 18th month of therapy onward. All those results are summarized in Table 2. The HIT-6 means at the seventh injection cycle and subsequent ones resulted always significantly lower than baseline (*P* < 0.05) and showed also a continuous, gradual decrease. Also SF-36 mental and physical scores were significantly lower than baseline at every injection session from the 18th months onward. ZUNG-A and ZUNG-D scores were not significantly different from baseline even if a gradual improvement was found. Only ZUNG-A score mean at the 33rd month of treatment was significantly lower than baseline (*P* < 0.05). In Figure 1, we reported the MNHD, AC, VAS score, HIT-6 score and SF-36 mental and physical scores means at every injection session with the corresponding 95% confidence intervals (CI). Secondary end-points changes VS baseline are reported in Table 3. The Student’s *t*-tests performed outpointed that the means of all explored parameters were not significantly different between the seventh injection cycle and the last one (13th) (Table 4). The two-way analysis of variance performed showed no significant differences between the mean of the explored end-points in the different preventive treatments group for all explored parameters (data not shown).

**Table 2:** HI, AC, and visual analog scale for pain (VAS) score means at every time-point and relative 95% CI.

Injection number	1 (*n* = 90)	7 (*n* = 27)	8 (*n* = 21)	9 (*n* = 20)	10 (*n* = 18)	11 (*n* = 18)	12 (*n* = 15)	13 (*n* = 13)
Mean number of headache days over 30 days	0.98 ± 0.16	0.52 ± 0.34**	0.5 ± 0.27**	0.51 ± 0.3**	0.53 ± 0.3**	0.49 ± 0.31**	0.48 ± 0.3**	0.49 ± 0.29**
VAS	7.66 ± 1.56	3.57 ± 1.35**	3.52 ± 1.97**	3.55 ± 1.79**	3.44 ± 1.69**	3.61 ± 1.61**	3.27 ± 1.67**	3.31 ± 1.25**
AC	1.98 ± 1.69	0.53 ± 0.42**	0.5 ± 0.27**	0.48 ± 0.28**	0.53 ± 0.3**	0.47 ± 0.28**	0.49 ± 0.29**	0.49 ± 0.29*

*P < 0.05, **P < 0.01, significance refers to the result of one-way ANOVA followed by Tukey–Kramer post hoc comparison test. Every column is named with the number treatment cycle after the first injections. Numbers in brackets indicate patients.

**Table 3:** ZUNG-A, ZUNG-D, 6-items headache impact test (HIT-6), and SF-36 Mental and Physical scores at every time-point VS baseline.

Injection number	1 (*n* = 90)	7 (*n* = 27)	8 (*n* = 21)	9 (*n* = 20)	10 (*n* = 18)	11 (*n* = 18)	12 (*n* = 15)	13 (*n* = 13)
HIT-6	65.1 ± 6.24	60.04 ± 7.2*	58.52 ± 8.04**	57.85 ± 7.44**	57.22 ± 7.3**	58.28 ± 8.37*	57.2 ± 7.88**	57.15 ± 5.7*
SF-36P	43.51 ± 18.49	58.58 ± 23.32*	60 ± 22.47*	63.22 ± 21.16**	67.22 ± 16.93**	63.83 ± 21.71**	65.07 ± 16.7**	65.2 ± 14.53*
SF-36M	46.14 ± 21.3	61.55 ± 21.83*	63.9 ± 20.78*	67.25 ± 21.07**	68.39 ± 18.64**	67.4 ± 22.96*	69.12 ± 20.02*	69.13 ± 15.1*
ZUNG-A	42.19 ± 10.42	39.32 ± 8.51	37.19 ± 9.58	36.05 ± 7.05	35.28 ± 8.49	35.61 ± 7.47	33.2 ± 8.06*	34.69 ± 7.41
ZUNG-D	43.02 ± 11.38	41.18 ± 9.53	39.1 ± 9.58	38.45 ± 10.03	37.65 ± 10.83	38 ± 11.26	36.21 ± 11.29	36.23 ± 10.19

*P < 0.05, **P < 0.01, significance refers to the result of one-way ANOVA followed by Tukey–Kramer post hoc comparison test. Every column is named with the number of months after the first injections. Numbers in brackets indicate patients.

**Table 4:** Student’s *t*-test for the primary and secondary end-points at the 18th and the 36th month of treatment.

End-point	Mean comparison
Mean number of headache days over 30 daysMNHD	0.52 ± 0.34 VS 0.49 ± 0.29
Visual analog scale for pain	3.57 ± 1.35 VS 3.31 ± 1.25
AC	0.53 ± 0.42 VS 0.49 ± 0.29
ZUNG-D	41.18 ± 9.53 VS 36.23 ± 10.19
ZUNG-A	39.32 ± 8.51 VS 34.69 ± 7.41
SF-36M	61.55 ± 21.83 VS 69.13 ± 15.1
SF-36P	58.58 ± 23.32 VS 65.2 ± 14.53
6-items headache impact test	60.04 ± 7.5 VS 57.15 ± 5.7

*P < 0.05, **P < 0.01.

### Adverse Events

All treated patients had only mild side effects and none of them caused patients’ discontinuation from OnabotA injections. The most frequent AEs were transitory and localized in the injection sites, mainly due to the injection procedure rather than OnabotA effects. They were: erythema (7/90, 7.7%), injection-site edema (3/90, 3.3%) and itching (3/90, 3.3%). OnabotA-dependent AEs were: muscles weakness (3/90, 3.3%), headache (2/90, 2.2%), and transitory palpebral ptosis (1/90, 1.1%). Globally, the 13.3% of patients suffered for an AE. No correlation were seen between AEs and injection number: all AEs were equally distributed across all injection sessions (data not shown). All AEs are summarized in Table 5.

**Table 5:** Treatment AEs.

Adverse event	Number of patients (%)
Injection-site edema	3/90 (3.33)
Injection-site itching	3/90 (3.33)
Muscles weakness	4/90 (4.44)
Headache	1/90 (1.11)
Neck pain	1/90 (1.11)
Eyelid ptosis	1/90 (1.11)
Total	12/90 (13.33)

## Discussion

Chronic migraine complicated with MOH is a hard challenge for clinicians dealing with headaches, because of its high frequency, its clinical impact, and the enormous treatments failure rate (21). Poor efficacy and low adherence to prophylaxis treatments are the most relevant issues (22), and they could affect OnabotA too, as demonstrated in other conditions rather than CM (23). To assess OnabotA effectiveness over time, its mean effect through the injection cycles was analyzed. The linear regression model slopes outpointed a significant mean improvement for all outcomes and this seems to indicate a significant, continuous and stable, OnabotA action over time. The most remarkable improvements were observed in the first year of therapy (Figure 1), and the results are stable over time, as pointed out also by other authors (24–26). The improvements of explored outcomes found out with the linear regression models were further analyzed to check their impact on clinical practice, performing the one-way analysis of variance. Primary end-points were significantly lower than baseline at every injection session from the eighteenth month (seventh cycle) onward, indicating the persistence of OnabotA effects, even after years (Table 2). This has been confirmed for shorter periods of time by other authors (24–28), and by the low anti-OnabotA antibodies production showed in a recent meta-analysis (29). In our experience, pain intensity and frequency have a similar trend: between the first and the last injection cycle, VAS score had a reduction rate of 52%, while MNHD about 45%. This reduction in the MNHD and its trend are substantially reflected by the percentage of chronic migraineurs at every year of therapy: a bigger amelioration was seen after the first year, with a subsequent stabilization: this proposes once again the possible existence of placebo effect, especially during the first month of treatment (10). The reduction in AC is more relevant, showing a higher improvement than in other studies (24, 25, 28, 30). The reduction in AC indicates that OnabotA could be a useful therapy to reduce the analgesics overuse, even without a detox therapy (31). This is corroborated by the fact that MOH-sufferers have a similar trend, decreasing from the 97.8% of the beginning to the 59.32% after the first year. At the second year, a small worsening in their proportion was registered (61.9%), while at the end the proportion sets on 53.85%. The last three proportion were significantly different from the baseline, but not between them, confirming once again the stability of OnabotA effect after the first year of treatment (24, 25). Regarding the headache impact on patients’ quality of life, the HIT-6 score means decrease at every time-point, from the 18th months onward. The persistence of HIT-6 reduction over time has been shown by other authors, even if for a brief period (24, 28). A stable and long-lasting improvement is a desirable goal in the management of the third most-disabling condition worldwide in under 50 s (32, 33). Accordingly, physical and mental health status scores improved through injection sessions, as stated by the SF-36 physical and mental means scores, which were significantly higher at every time-point, compared with baseline (Table 3). Similar to long term experiences in other therapeutic indications (such as several kinds of cervical dystonia), a majority of patients comply with repeated treatment because it provides a good and stable effect over time (34). Notably, this result was achieved despite the patients we studied had high values of MNHD, AC, VAS, and HIT-6 scores at baseline, a long migraine history and a high comorbidity rate (82%). Unlike previously studied outcomes, ZUNG-A and ZUNG-D scores means did not change significantly from baseline, confirming our previous findings (15). OnabotA efficacy on anxious and depressive symptoms, associated or not with chronic pain, is a matter of debate and a growing amount of literature is dealing on it, with disagreeing results. Two randomized placebo-controlled trials revealed positive effects of OnabotA in depressed patients: Wollmer and colleagues demonstrated that OnabotA injections in the glabellar region improved the Hamilton depression rating scale (HDRS) in a cohort of 15 depressed patients (35), while Finzi and Rosenthal obtained similar results using the Montgomery–Asberg Depression Rating Scale (MADRS) (36). Hence, Hexsel and colleagues found a significant improvement in Beck depression inventory (BDI) score after OnabotA injections in the corrugator muscle of major-depressed patients (37). However, Aydinlar et al. found no significant changes in the 21-items depression, anxiety, and stress (DASS-21) score after 1 year of therapy (38), and Maasumi et al. revealed no significant changes in the median of patient health questionnaire-9 (PHQ-9) (39). Despite the question about the OnabotA effects on depressive symptoms remains open (40), in our observations no improvements on depressive or anxiety symptoms were observed using ZUNG-A and ZUNG-D scores. The small sample sizes and the different questionnaires administered limit the reliability of every comparison. As far as it concerns the OnabotA safety profile, it is still confirmed even after 3 years of therapy. The lack of drop-outs due to AEs and the majority of them due to the injection procedure, rather than OnabotA-related events, confirms its high tolerability (24–28, 34). The combination of OnabotA safety and effectiveness gives reasons to its high and consistent adherence also in the long term, with a discontinuation rate much lower than other prophylactic drugs, around 50% already after only 6 months of treatment (41). Neither tolerability nor efficacy were analyzed comparing 155 U and the 195 U dosages, because the patients who underwent the “follow the pain” strategy were only 8 and in isolated cases, strongly limiting the reliability of any statistical analysis. The co-existence of another preventive treatment did not affect OnabotA response: from the two-way analysis of variance no significantly differences were found in the means of the explored parameters between the different class A preventive drugs for migraine (data not shown), even if the small number of patients in the last injections limits the reliability of this analysis. This study has some limits: the lack of a control group makes impossible to quantify the placebo effect. Placebo response is usually high in migraine and even higher during OnabotA treatment (42–44), but the uncertain data regarding placebo persistence after 18 months and the stable ameliorations after that time-point suggest a pharmacological effect rather than placebo. A strong reduction in the number of patients was seen from the 1st to the 13th injection: 24 patients changed preventive treatment other than OnabotA and were excluded from the computation, one tried a muscular electric stimulator, 14 patients were considered as drop-outs and 38 are still on treatment.

## Conclusion

OnabotulinumtoxinA is an effective and safe treatment for CM complicated with MOH as confirmed by our experience in the short and long term. OnabotA did not show any incoming tolerance and its effectiveness was confirmed to be long-lasting, generating stable improvements in headache symptoms and patients’ quality of life. These findings strongly contribute to support the benefits of long term regular administrations of OnabotA injections for several years in order to maintain a consistent migraine relief. A recent Italian survey outpoints that one-third of clinicians discontinue OnabotA if the benefits persist for at least 6 months, often postponing the injections for more than 3 months (44). Our experience demonstrated that OnabotA can be administered continuously over several years, according to the PREEMPT protocol, thanks to its persistent efficacy, its good AEs-related profile and taking into account a potential worsening of the symptoms after its suspension (15).

## Ethics Statement

This study was carried out in accordance with the recommendations of Provincial Ethical Committee of Modena with written informed consent from all subjects. All subjects gave written informed consent in accordance with the Declaration of Helsinki. The protocol was approved by the Provincial Ethical Committee of Modena (protocol number: 334/15).

## Author Contributions

LAP, SG, MC, LP, AN, and PM drafted the manuscript. CB made statistic calculations and contributed to draft the manuscript. LP and SG conceived the study. All authors read and approved the final manuscript.

## Conflict of Interest Statement

LP and SG received grants and travel honoraria from Allergan. PM received honoraria, travel bureau, research grant, and advisory board from ACRAF, Allergan, Amgen, Electrocore, Elytrapharma. AN received honoraria, travel bureau, research grant, advisory board from Allergan. The authors declare that the research was conducted in the absence of any commercial or financial relationships that could be construed as a potential conflict of interest. The reviewer CD declared a shared affiliation, with no collaboration, with several of the authors, PM and AN, to the handling Editor.

## Abbreviations

AC, analgesic consumption; BAI, Beck anxiety inventory; BDI, Beck depression inventory; CI, confidence intervals; CM, chronic migraine; DB, double-blind; DASS-21, 21-items depression, anxiety and stress scale; HDRS, Hamilton depression rating scale; HIT-6, six item headache impact test; ICHD-III-beta, International classification of headache disorders-third edition, beta version; U, Units; MADRS, Montgomery-Asberg depression rating scale; MIDAS, migraine disability assessment questionnaires; MNHD, mean number of headache days over 30 days; MSQ, migraine-specific quality of life questionnaire; PHQ9, 9-items patient health questionnaire; PHQDM, Patient Health Questionnaire depression module; PREEMPT, Phase III Research Evaluating Migraine Prophylaxis Therapy Trail; MOH, medication overuse headache; OnabotA, OnabotulinumtoxinA; SF-36, short form health survey questionnaire; VAS, visual analog scale for pain; ZUNG-A, Zung self-rating anxiety scale; ZUNG-D, Zung self-rating depression scale.
