# Addendum: Long-term Treatment Benefits and Prolonged Efficacy of OnabotulinumtoxinA in Patients Affected by Chronic Migraine and Medication Overuse Headache over 3 Years of Therapy

**DOI:** 10.3389/fneur.2018.00641

**Published:** 2018-08-07

**Authors:** Simona Guerzoni, Lanfranco Pellesi, Carlo Baraldi, Michela Maria Cainazzo, Andrea Negro, Paolo Martelletti, Luigi Alberto Pini

**Affiliations:** ^1^Headache and Drug Abuse Research Centre, Policlinico Hospital, Department of Diagnostic Medicine, Clinical and Public Health, University of Modena e Reggio Emilia, Modena, Italy; ^2^Regional Referral Headache Centre, Sant'Andrea Hospital, Department of Clinical and Molecular Medicine, Sapienza University, Rome, Italy; ^3^Center for Neuroscience and Neurotechnology, Department of Biomedical, Metabolic and Neural Sciences, University of Modena e Reggio Emilia, Modena, Italy

**Keywords:** chronic migraine, OnabotulinumtoxinA, long-term treatment, quality of life, tolerance, headache, medication overuse headache

In the original article, there was a mistake in the title for **Table 2**, as published. The correct title should be *MNHD, AC, and visual analog scale for pain (VAS) score means at every time-point and 95% CI*.

Similarly, in the sentence “HI, AC, and VAS score were considered as the primary end-points of the study” the acronym HI was used. It has now been changed to MNHD. We also omitted the formula used to calculate the MNHD. A correction has been made to Materials and Methods, sub-section Procedures, Paragraph 1. The corrected paragraph appears below:

During every injection session, the mean number of headache days over 30 days (MNHD), the mean number of abortive medications taken every day (analgesic consumption- AC) and the mean value of the visual analog scale for pain (VAS) score were collected from the headache diaries. MNHD, AC and VAS score were considered as the primary end-points of the study. Mean Number of Headache Days over 30 Days (= Number of headache days in the 1^st^ month after injection + Number of headache days in the 2^nd^ month after injection + Number of headache days in the 3^rd^ month after injection / 90).

We created a CONSORT chart to better explain the number of drop-outs seen during the study. This has been added as Figure 2 and is cited in Results, sub-section Demographic Analysis and Drop-outs, which is available below:

**Figure 2:**
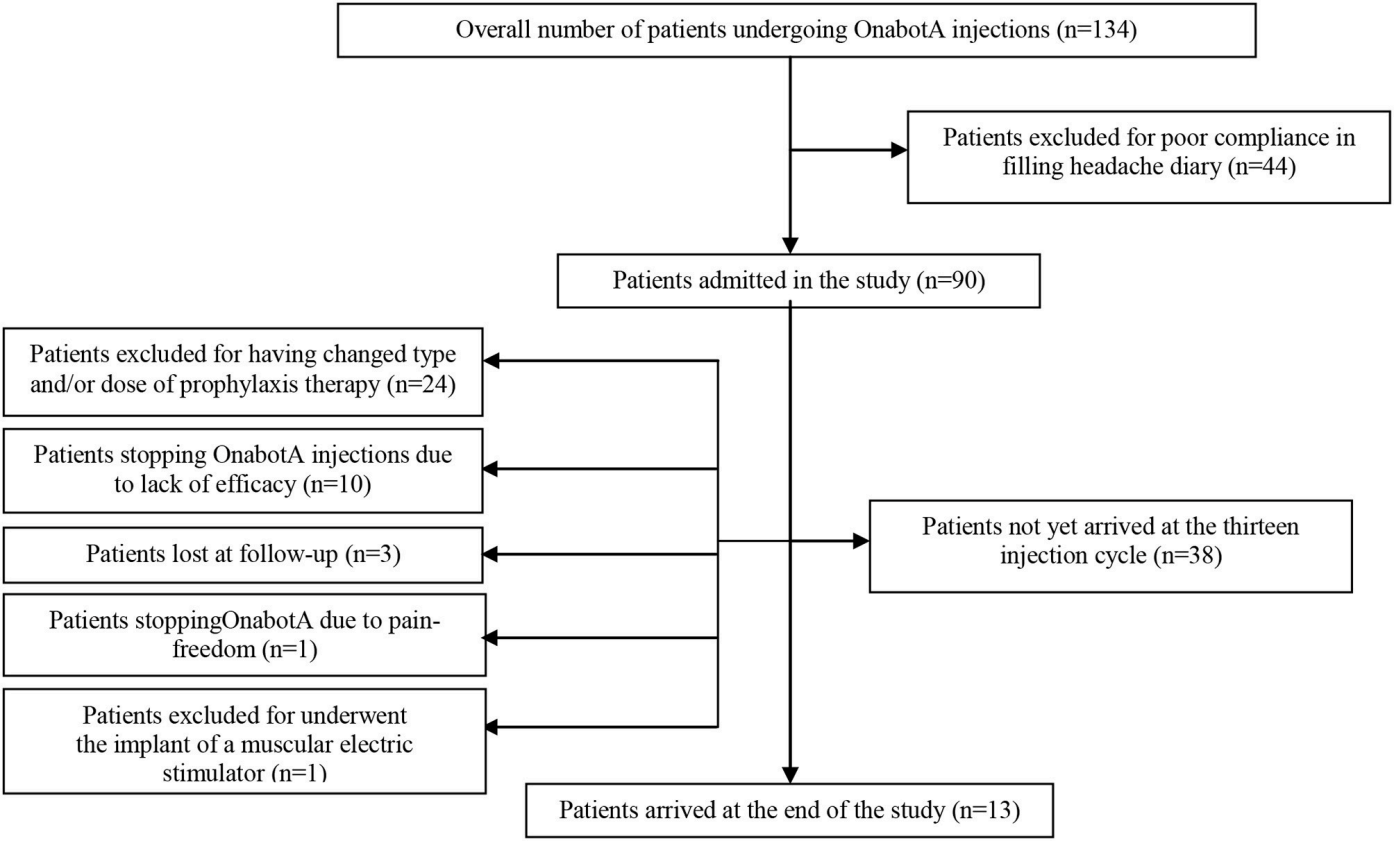
Patients and drop-outs.

The analyzed sample was composed by 90 patients, 14 men and 76 women, aged between 35 and 65 years (mean ± SD = 45.21 ± 10.12). The most overused drugs were triptans (71/90-78.89%) followed by non-steroidal anti-inflammatory drugs (NSAIDs) (41/90-45.56%), whilst only 3 patients overused combination drugs (3-3.33%). Oral drugs were taken by the 88% of patients, the 20% used also intramuscular drugs and the 15% used also rectal formulations. Thirty-four patients used a first class preventive treatment other than OnabotA: 4 used anti-hypertensive drugs, 15 antidepressants and 15 antiepileptics. No patients took simultaneously two first class preventive treatments. Patients who took anti-hypertensive drugs stopped them before the 7^th^ injection cycle, due to inefficacy (3 patients) and one adverse event (hypotension), so data from these patients were not pooled in the two-way analysis of variance. Eight patients underwent the 195 U treatment during no more than one injection cycle each. Eighty-eight out of 90 patients (97.8%) fulfilled the diagnostic criteria for chronic migraine at the beginning of the study. After the first year of treatment patients suffering for CM were 37 out of 59 (62.72%), becoming the 66.67% at the second year (14 out of 21 patients) and the 53.85% at the 3^rd^ year (7 out 13 patients). The proportion of chronic migraineurs at the baseline is lower than the ones at the 1^st^, 2^nd^ and 3^rd^ year. Those ones were not significantly different (Fisher's exact test- data not shown). All 88 chronic migraineurs at the beginning were also considerable as MOH-sufferers. After the first year of therapy their percentage decreased to the 59.32% (35 out of 59 patients). At the second year the proportion of MOH-sufferers increased at 13 out of 21 (61.9%) and at the third year became of 7 out of 13 patients (53.85%). MOH-sufferers proportion at the baseline was significantly higher than the ones at future time-points, but no significant differences were found between them (Fisher's exact test, data not shown). Of the 90 patients enrolled, 24 changed the dose and/or type of preventive treatment other than OnabotA due to side effects and, even if they continued OnabotA injections, further data were not pooled in the analysis. One patient tried a muscular electric stimulator without consulting physicians and her data from that moment onward were not pooled in the analysis. Globally, 14 patients stopped OnabotA injection during the observation (14/90-15.56%): one patient decided to stop treatment because she was almost pain-free, three patients were lost at follow-up and ten patients discontinued OnabotA due to lack of efficacy. No drop-outs were caused by OnabotA-related AEs. Globally, only patients who discontinued OnabotA treatment because of lack of benefit and those ones who were lost at follow-up were considered as drop-outs, giving an overall number of drop-outs of 13/90 (14.44%). A CONSORT flow-chart was added for graphically explained the drop-outs reasons (Figure 2).

The authors apologize for these errors and hope the clarification aids readers' understanding of the article. These changes do not change the scientific conclusions of the article in any way.

## Conflict of interest statement

LP and SG received grants and travel honoraria from Allergan. PM received honoraria, travel bureau, research grant, advisory board from: ACRAF, Allergan, Amgen, Electrocore, Elytrapharma, AN received honoraria, travel bureau, research grant, advisory board from Allergan. The remaining authors declare that the research was conducted in the absence of any commercial or financial relationships that could be construed as a potential conflict of interest.

